# 

**Published:** 1852-08

**Authors:** 


### CONTENTS

### OF NO. II.

---

### ORIGINAL COMMUNICATIONS.

### ESSAYS AND CASES.

ART. I.—A Case of Spinal Irritation, attended with loss of Vision; Recovery. By CHARLES FISHBACK, M. D., of Shelbyville, Ind. - - - - - - - - 93

ART. II.—Two Cases of Fatal Injury of the Head. By ROBERT DURRETT, M. D., Resident Graduate of the Louisville Marine Hospital, for 1851. - - - - - - 99

ART. III.—A Case of Glanders in the Human Subject. By JOSIAH T. WATTS, M. D., of Enterprise, Miss. - 101

ART. IV.—On Vital Organic Chemistry. By C. CALDWELL, M. D., Late Professor of the Institutes of Medicine in the University of Louisville. - - - - - - 103

### BIBLIOGRAPHICAL NOTICES.

ART. V.—The Vital Statistics and Sanitary Condition of Memphis, Tennessee, an Anniversary Address, delivered by appointment, before the Memphis Medical Society, on the 5th of February, 1852. By GEO. R. GRANT, M. D. 134

ART. VI.—A History of Epidemic Cholera, as it appeared at the Baltimore City and County Alms-House, in the Summer of 1849, with some remarks on the Medical Topography and Diseases of this Region. By TH. H. BUCKLER, Physician to the Baltimore City and County Alms-House. 138

ART. VII.—The Annual Address to the Graduating Class in the Medical Institution of Yale College, January 15, 1852. By ALVAN TALCOTT, M. D., in behalf of the Board of Examiners. - - - - - - - - - - 140

SELECTIONS FROM FOREIGN AND HOME JOURNALS.

Cases Illustrating the Localization of Valvular Lesions of the Heart. Reported in the Buffalo Medical Association, by Austin Flint, M. D., - - - - - - 141
On Blistering in Gleet. - - - - - - - - - 150
On the Hydrated Peroxide of Iron and Magnesia as Antidotes in Poisoning with Arsenic. - - - - - - - 152
On the Treatment of Cancer by the Lactate of Iron taken by the Mouth and injected into the Veins. - - - - 153
Morbid Habit of Swallowing Hair; Prolonged sojourn of the foreign bodies in the gastro-intestinal canal. Evacuation of Packets of Hair by vomiting and alvine dejections. - 156
Deaths from Chloroform. - - - - - - - - 157
On Chloroform as an Emmenagogue. - - - - - - 158
On the Nutrition of Muscles during their Contraction. - - 160
A Case of Hydrophobia in the South of France. - - - - 162
Quinine in Continued Fever. - - - - - - - 164
On the Respiration of the Alligator, and Mrs. Willard's Theory of the Circulation. - - - - - - - - 166
The Cause and Prevention of Death from Chloroform, - - 169
Improvements in Modern Surgery. - - - - - - 173

ORIGINAL INTELLIGENCE.

Health of Louisville, - - - - - - - - - 177
Cholera, - - - - - - - - - - - 177
Circular, - - - - - - - - - - - 178
Dr. Grimes' Address, - - - - - - - - 181
Encouragement of Quackery in Georgia, - - - - - 183
New Medical Books. - - - - - - - - - 184

